# On the Obstetric Practice

**Published:** 1816-05

**Authors:** Richard Edgell

**Affiliations:** Bristol.


*For the London Medical and Physical Journal.*

### *On the Obstetric Practice;* by RICHARD EDGELL, M.D. of Bristol.

I WAS much astonished at reading, in your Number for March, in Dr. Kinglake's reply to Messrs. Wayte and Atkinson on Obstetric Practice, some observations which appear to me to have been made under a misconception of the subject. I have been some years engaged in extensive *man*-midwifery practice, and may, therefore, be permitted to hazard an opinion on the practical accuracy of Dr. Kinglake's deductions. With the general controversy I have nothing to do—my object is to attempt to show that Dr. K. has been misinformed.

1st.—With regard to the placental presentation;—Dr. Kinglake says, "if my information be correct, it is not risking too much to say, that there is not more than one practitioner in a thousand, in any age, in any country, that has ever met with an instance of it."—In contradiction to this I have to observe, that our most eminent writers on midwifery mention it as not of unfrequent occurrence; and I would particularly call his attention to a most useful practical work in corroboration of my assertion, namely, Rigby on Uterine Hæmorrhage; in addition to which, I beg to state, that attachment of the placenta to the os uteri has not been unfrequent in Bristol. It has happened to myself, and to every extensive practitioner in midwifery with whom I have had any conversation on the subject; and instances of death by flooding from placental presentation have come to my knowledge, where the unhappy patient has been under the direction of ignorant or unskilful practitioners. I believe it was an aphorism of the late Dr. Clarke, that no woman should die of hæmorrhage if the accoucheur be called early, and certainly my observation as yet induces me to form the same conclusion. Dr. Kinglake's supposition that a case of placental presentation, if left to Nature, would do well, and that the uterine contraction that would detach the placenta would also advance the head of the fœtus sufficiently to restrain the hæmorrhage by its pressure, is worthy of refutation, as it may otherwise be acted upon by some inexperienced practitioner, which would, in most cases, cause the death of the patient. The Doctor does not appear to have given his practical conclusions that degree of attention they demand; for he has evidently mistaken *dilatation* for *contraction*, as it is by the dilatation of the os uteri that the placenta becomes detached, and the most dangerous cases of flooding flooding are those in which no contraction at all takes place. The membranes containing the waters would in most cases prevent the head of the fœtus advancing, or the uterus from contracting sufficiently to restrain the hæmorrhage; and he will find, by the facts stated by Mr. Rigby, which stand uncontroverted, that hæmorrhages occasioned by a partial separation of the placenta, by other causes than attachment to the os uteri, will even require the rupture of the membranes to cause the contraction of the uterus sufficient for putting a stop to the hæmorrhage by its pressure on the body of the infant.

2dly.—With respect to giving assistance in cases of rigid os uteri. Here the Doctor has not been sparing of hard words; but declamation will not bear down facts. It is well known that cases of severe and protracted labour have been expedited or relieved by "*intermeddling*" with the lancet or catheter; and where there is the probability of preventing a patient from suffering several hours of torment, or even death, from rupture of the uterus or bladder, such intermeddling should not be designated "mischievous or useless," &c.

3dly.—In severe cases of hæmorrhage after delivery, the Doctor asserts, "no stimulant medicines would be admissible, no manual aid could be usefully offered." As a general rule, I believe he is right; but I beg leave to assure him I have been concerned in cases where I am of opinion that, if I had not, by the local application of cold, or even the introduction of the hand, and in others by the constant exhibition of "stimulant medicines," restrained the flooding, I should have lost my patients.

4thly.—In reply to Mr. Atkinson, Dr. Kinglake puts a stop, as he thinks, to all further discussion, by the following extraordinary assertions:—"*It has been ascertained, to an extent that sets all question at rest on the subject, that medical practitioners in full midwifery employ during upwards of thirty years have never met with an unnatural presentation, have never had an occasion for using an instrument, and have always found the natural efforts equal to all the exigencies of salutary parturition.*" But these thirty-year gentlemen have not condescended to tell us how many of their patients died undelivered. I remember well that a Somersetshire practitioner, in answer to a general observation that no woman should die undelivered, said, "that is very pretty indeed, but how can we help it? I have had many women die undelivered."

Believe me, gentlemen, I have no unworthy motive for this this communication: it is hastily elicited by a perusal of Dr. Kinglake's paper, which, coming from respectable authority, if suffered to go unanswered, may be the cause of much mischief.

*March*, 1816.

---

*For the London Medical and Physical Journal.*

*On Cutting off the Tails of Leeches;* by S. M.

